# Experts’ perceptions on the use of visual analytics for complex mental healthcare planning: an exploratory study

**DOI:** 10.1186/s12874-020-00986-0

**Published:** 2020-05-07

**Authors:** Erin I. Walsh, Younjin Chung, Nicolas Cherbuin, Luis Salvador-Carulla

**Affiliations:** 1 Centre for Research on Ageing, Health and Wellbeing, Research School of Population Health, College of Health and Medicine, Australian National University, Canberra, Australia; 2 PHXchange (Population Health Exchange), Research School of Population Health, College of Health and Medicine, Australian National University, Canberra, Australia; 3 Centre for Mental Health Research, Research School of Population Health, College of Health and Medicine, Australian National University, 63 Eggleston Road, Acton, ACT 2601 Australia

**Keywords:** Visual analytics, Expert experience, Complex data analysis, Mental healthcare systems, Evidence-informed decision-making, Co-development

## Abstract

**Background:**

Health experts including planners and policy-makers face complex decisions in diverse and constantly changing healthcare systems. Visual analytics may play a critical role in supporting analysis of complex healthcare data and decision-making. The purpose of this study was to examine the real-world experience that experts in mental healthcare planning have with visual analytics tools, investigate how well current visualisation techniques meet their needs, and suggest priorities for the future development of visual analytics tools of practical benefit to mental healthcare policy and decision-making.

**Methods:**

Health expert experience was assessed by an online exploratory survey consisting of a mix of multiple choice and open-ended questions. Health experts were sampled from an international pool of policy-makers, health agency directors, and researchers with extensive and direct experience of using visual analytics tools for complex mental healthcare systems planning. We invited them to the survey, and the experts’ responses were analysed using statistical and text mining approaches.

**Results:**

The forty respondents who took part in the study recognised the complexity of healthcare systems data, but had most experience with and preference for relatively simple and familiar visualisations such as bar charts, scatter plots, and geographical maps. Sixty-five percent rated visual analytics as important to their field for evidence-informed decision-making processes. Fifty-five percent indicated that more advanced visual analytics tools were needed for their data analysis, and 67.5% stated their willingness to learn new tools. This was reflected in text mining and qualitative synthesis of open-ended responses.

**Conclusions:**

This exploratory research provides readers with the first self-report insight into expert experience with visual analytics in mental healthcare systems research and policy. In spite of the awareness of their importance for complex healthcare planning, the majority of experts use simple, readily available visualisation tools. We conclude that co-creation and co-development strategies will be required to support advanced visual analytics tools and skills, which will become essential in the future of healthcare.

**Graphical abstract:**

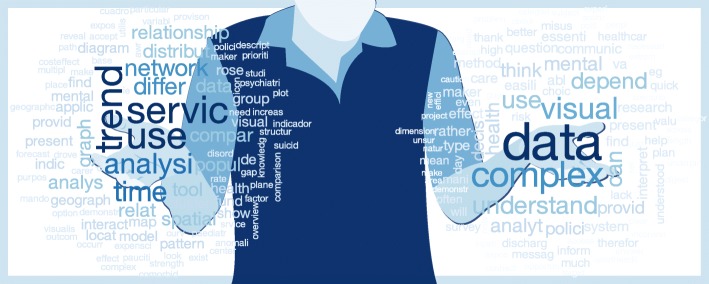

## Highlights

- Visual analytics are useful decision-making aids for complex healthcare systems
- Two thirds of experts understand the importance of visual analytics
- Over half of experts expect more advanced visual analytics tools
- User-friendliness is key to expert engagement with visual analytics
- Co-development of future visual analytics tools may address low expert uptake

## Background

In order to monitor, evaluate, and plan for the future of mental healthcare systems, health experts including policy-makers require a deep knowledge of a constantly changing landscape of service distribution, management, demand, and outcomes. This is a vital challenge given the high-stakes nature of healthcare decision-making [1]. Understanding healthcare systems is important to health experts for their decision-making in policy, funding, and quality healthcare delivery. This requires reliable evidence, and the growing availability of healthcare data constitutes important evidence for better decision-making.

Visual analytics has great potential to develop evidence from healthcare data of increasing volume and complexity and support evidence-informed decision-making [2]. Visual analytics refers to the science of analytical reasoning facilitated by interactive visual interfaces [3]. It combines automated algorithms (e.g., machine learning) with visualisation techniques for human interaction and cognition in the process of data analysis. Data visualisation is the graphical representation of data, undertaken to illustrate relationships and patterns. It can be used to draw out tacit knowledge that can be incorporated into expert knowledge to improve the decision-making process [4]. Thus, visual analytics capitalises on complex data, enabling hypothesis generation, hidden pattern identification, interest expression, insight in data, evidence development, and communication for action. This involves missing value imputation, pattern identification and prediction, as well as statistical description and inference.

Individual or combined visualisations such as histograms, line graphs, bar charts, scatter plots, networks, and/or geographical maps are widely used to provide overviews, descriptions, and statistical summaries of data [5]. Visual analytics also plays an important role in exploratory and predictive analyses for complex and nonlinear pattern information to support actionable decision-making (e.g., policy changes). The application of visual analytics as a tool for complex data analysis and decision support can be found in healthcare. Examples include a cohort clustering analysis using disk-like visualisation in public healthcare [6], and an associative service pattern analysis using grid map visualisation in mental healthcare [7]. However, a lack of understanding, availability, development, and application of visual analytics methods answering complex questions persists in the process of evidence development and decision-making [8, 9].

In mental healthcare systems research and policy, there is a critical need to study effective ways of developing and using visual analytics to support the analysis of complex data for decision-making [9]. This involves methodological development of visual analytics methods with experts to allow appropriate understanding, reasoning processes and skills in discovering complex information, and generating evidence-informed knowledge. A recent review has examined mental health expert usage of visual analytics in published studies [9]. However, in face-to-face interviews with Australian health experts, only one quarter reported referring to academic journals when gathering evidence for policy with the majority turning to other organisations or colleagues [10]. This indicates that experience and knowledge among health experts may not be the same as that contained in scholarly research publications. This may contribute to an opportunity-cost arising from a disconnect between the availability of advanced visual analytics tools in the scholarly literature - which can keep pace with complex data analysis - and their actual utilisation in decision-making.

There is currently a lack of information on the experience of experts engaging with visual analytics and their expectations for visual analytics tools that may be developed for better data analysis and decision-making processes. This may be because literature searches are largely insensitive to this information, which is better revealed by qualitative and self-report measures (e.g., [11]). Surveys and interviews are undertaken in such circumstances to explore the extent and nature of a research activity, prior to larger scale literature review or data collection [12]. This approach can be the first step toward an ongoing process where domain experts are involved in development and iterative improvement of a system or tool, known as co-development [13]. Co-development has demonstrated benefits in healthcare decision-making [4], and in the current setting may leverage insight to produce more useful visual analytic methods and equip health experts with skills for applying novel visualisation techniques. To support the application of a co-development approach, we need to improve our understanding of how healthcare experts engage with and perceive complex data visualisations.

The aims of this exploratory study were: (1) to examine the real-world experience that experts in mental health have with visual analytics; (2) to investigate how well widely available visual analytics techniques meet their needs; and (3) to codify their understanding and expectations to establish priorities for the future development of visual analytics tools of practical benefit to mental healthcare policy and decision-making. In order to guide further research, we also examine whether experience, needs, and priorities for future development differ based on the expert’s current working area (policy, research, or service delivery) and the degree of data complexity. To our knowledge, this is the first study to date to explore expert understanding and expectations of visual analytics for complex mental healthcare system planning.

## Methods

### Participants

Participants were sampled from an expert pool of policy-makers, health agency directors, and researchers with direct experience of using visual analytics tools for complex mental healthcare systems planning. This was achieved via snowball sampling through two networks in Australia and in Europe that included experts of mental health policy research and in mental health economics:

- The VIDEA (VIsual and DEcision Analytics) Lab - a visual analytics service research hub based at the Australian National University; and
- The European PECUNIA project (ProgrammE in Costing, resource use measurement and outcome valuation for Use in multi-sectoral National and International health economic evaluAtions) consortium.

A total of 151 email invitations were sent at the end of May 2019. Forty respondents completed the survey (27% response rate from 151 invitations). Participants included chief executive officers, professors, and international project directors. They had a median of 15 years (1–55 year range, Standard Deviation = 12 years) working experience dealing with mental healthcare systems data. The most common areas of work were research (32%), policy (25%), and service provision (10%). Others reported working in the area of health economics (7.5%), defence (2.5%), communication (2.5%), advocacy (2.5%), and teaching (2.5%). The rest (15.5%) was either unspecified or fell within the broad category of ‘health’. Respondents were mostly based in Australia (55%) with others being located in Europe (Spain 20%, United Kingdom 2.5%, Denmark 2.5%, France 2.5%, Austria 2.5%, Switzerland 2.5%, and the Netherlands 2.5%). Eleven percent of respondents did not specify their country of origin.

### Questionnaire

Respondents followed a link from invitation emails to complete an online questionnaire via the Qualtrics survey platform. Survey questions are presented alongside results, and the full questionnaire is included in the supplementary materials. In order to establish expert experience, data challenges, and visual analytic needs, questions were divided into three sections: (1) Demographics (role, area of work, years of expertise, institution and country); (2) Mental healthcare systems data (data types, aspects, analyses, outcomes, and complexity); and (3) Visual analytics (experience, preferences, need for new or advanced tools, and willingness to learn new techniques). The questionnaire concluded with an open-ended invitation for any further comments. Data collection was closed on 31st of July, 2019.

### Data analysis

All analyses were undertaken in R [14], using the packages ‘tm (v0.6–2)’ [15], ‘stringr (v1.10)’ [16], ‘LSAfun (v0.5.1)’ [17], and ‘ggplot2 (v 2_3.1.0)’ [18].

Quantitative analysis consisted of descriptive statistics (frequencies and ranks) and Chi-square (χ^2^) analyses. In order to quantify expert experience and needs, categorical responses were explored in terms of descriptive statistics (frequencies and ranks). Chi-square (χ^2^) analysis was undertaken to explore whether or not expert expectations and requirements for visual analytics differed on the basis of work area and perceived data complexity. This analysis can indicate whether or not the differences should be taken into account in future work. To account for unbalanced cell sizes and problems arising from cells with < 5 members [19], the sparser responses in categorical variables were collapsed into ‘other’ categories. For these analyses, area of work was collapsed into a categorical variable (policy, research, and service provision/other). Degree of data complexity was treated as an ordinal variable due to strong negative skew (toward high complexity) and constrained rating scale (1–5). Visualisation techniques that respondents have the most experience working with were collapsed into categories of basic graphs or charts, geographical maps, and ‘other’. Monte Carlo simulation for significance [20] at 10000 replicates was undertaken for significance testing.

Power analysis was also undertaken, using the ‘pwr package’ in R. We chose a conservatively large power requirement to account for the increased likelihood of type II error (failing to reject a null hypothesis which is true) given the comparatively small sample size [21]. Alpha (α; evidence strength before one rejects the null hypothesis) was set at 0.05, sample size (*n*) was set to our available sample of 40, and degrees of freedom were set based on the number of categories in both dependent and independent variables. Power calculations for χ^2^ indicate that our planned analyses were sensitive to large or medium-to-large sizes only [22]. Specifically, the minimum detectable effect size with our data is 0.49 (with *n* = 40, two degrees of freedom, alpha = 0.05, power = 0.8). Inference based on statistical significance under these conditions can be meaningful if a large effect size is present, but should be interpreted with caution because it may miss smaller trends in the data [19].

Open-ended responses were explored through qualitative synthesis and text mining. For text mining, corpora were created from open-ended responses by removal of stop words, case, punctuation and stemming (e.g., “service”, “services”, become “service*”) using Porter’s algorithm [23]. The asterisk symbol (*) indicates word truncation, e.g., ‘service*’ corresponds to ‘service’, ‘service*s*’, ‘service*d*’, etc. Word frequencies were used to identify key terms. Neighbourhood analysis was undertaken to find the terms conceptually most related to “health”, while taking into account baseline word frequency in the English language.

## Results

### Qualitative analysis

#### Mental healthcare systems data

**Fig. 1:**
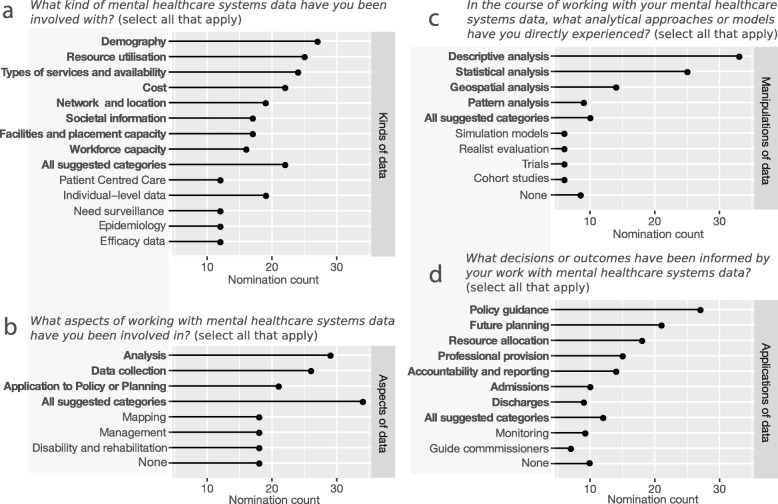
Participant experience with mental healthcare systems data. This figure summarises responses to multiple-choice questions in Section 2 of the questionnaire. In a, b, c, and d, bold label indicates provided options as suggested categories, lighter weight indicates categories entered by respondents under “Other: please specify” of each question

Participant experience with mental healthcare systems data is summarised in Fig. 1. Participants were most involved with demography and resource utilisation (Fig. 1a). They tended to be involved across the stages of data collection, analysis, and application to planning (Fig. 1b), with a focus on descriptive analysis (Fig. 1c). The most common outcomes of their involvement were policy guidance and future planning (Fig. 1d).

In open-ended responses to “*What have you used analytical approaches in your mental healthcare systems data for?*” the most frequent term was “service”, and the closest correlate of the concept of “health” was “cost-effect*”. This indicated a general focus on managing healthcare service planning and delivery within budgetary constraints. On a 5-point Likert scale, participants rated their data as very complex (33%) or complex (22.5%), indicating that mental healthcare systems data was predominantly complex. The highest ranked contributor to this complexity was the data structure (e.g., complex interactions between resources, services, patients, and agents within or between practices and catchments) as seen in Table 1.

**Table 1:** Participant ranking on the source of complexity in their data

	Overall Rank	Number of participants assigning each source of complexity at this particular rank (1–9)								
Source of complexity		1	2	3	4	5	6	7	8	9
Structure (complex = more nested elements)	1	4	5	4	6	4	4	3	–	–
Variety (complex = multiple data types)	2	3	4	5	7	3	6	–	2	–
Relationships (complex = more interaction between elements)	3	4	5	7	1	2	5	4	1	1
Number of variables (complex = more measures)	4	4	3	3	6	3	2	2	6	1
Uncertainty and ambiguity (complex = more uncertain)	5	6	4	2	2	3	1	3	7	2
Contributors (complex = larger number of individuals or data points)	6	3	3	4	1	6	3	4	4	2
Abstraction (complex = further away from raw measures)	7	3	3	1	3	2	2	10	2	4
Size (complex = larger number of individuals or data points)	8	2	1	2	2	4	3	1	6	9
Difficulty of prediction or forecasting (complex = more difficult)	9	1	2	2	2	3	4	3	2	11

Note. The rank, ‘1’ is the most central to their personal definition of data complexity, and ‘9’ is the least central. Numeric columns indicate how many participants ranked each source of complexity at that particular rank. ‘Overall rank’ indicates overall rank across participants, and it was calculated by summing these values and ordering source of complexity from the smallest value (globally the highest rank) through the largest value (globally the lowest rank)

#### Visual analytics

**Fig. 2:**
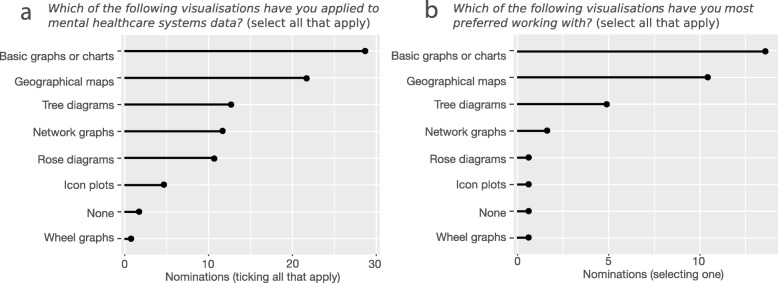
Participant experience with visual analytics. This summarises responses to multiple-choice questions in Section 3 of the questionnaire

Sixty-five percent of participants indicated that visual analytics is important (rather than neutral or unimportant) to their field. Participants’ experience with visual analytics is summarised in Fig. 2. Based on their analytical experience in Fig. 1c for descriptive, statistical, and geospatial analyses, basic graphs and geographical maps were identified as the most commonly applied and preferred visualisations (Fig. 2a and b). In open-ended responses to “*What did you use this visualisation method for?*” the most frequent terms were “trend” and “service*”. The closest correlated terms to the concept of “health” were “hotspot” (a form of visualisation) and “coplot” (software for producing visualisations [24]), indicating a tendency to use visualisations to track spatial or temporal trends.

#### Current experience and needs

**Fig. 3:**
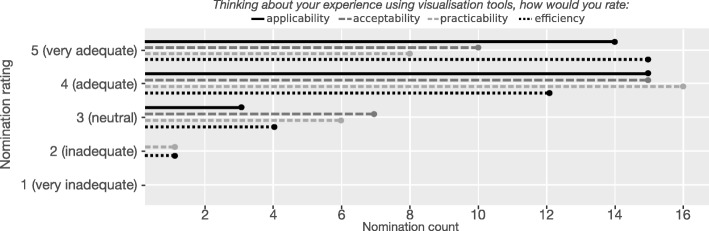
Participant rating of visualisation tools in terms of applicability, acceptability, practicability and efficiency

Figure 3 depicts overall participant ranking of their experience with visualisation methods across a set of criteria as defined in the questionnaire: applicability (the degree to which the visualisation is meaningful for analysis and subsequent decisions); acceptability (user-friendliness and likelihood of uptake); practicability (ability to effectively implement and interpret); and efficiency (capacity to clearly summarise large and complex data or model results). Participants who experienced visualisation methods rated these criteria primarily as “very adequate” or “adequate”, and none rated them as inadequate for understanding data in general. However, only 25% indicated current visual analytics approaches were adequate (rather than neutral or inadequate) to inform healthcare decisions. Given the options ‘*yes’*, ‘*maybe’* and ‘*no’*, 55% indicated that more advanced visual analytics tools were needed for their data analysis, and a majority of participants (68%) were willing to learn new visual analytics approaches.

In responses to a final open-ended invitation for further comments, the most frequently used terms were “data”, “complex”, and “understand”. The closest correlated terms to the concept of “health” were “maker” and “system”, which are indicative of a focus on expert knowledge integration with healthcare data visualisation. An exemplified response is:

“*Visual analytics is essential to understand complex systems such as mental health care data. I think it is necessary to deep into this area to improve user-friendliness and to provide a better overall understanding by political decision makers.*”

The closest correlated terms to the concept of “visual*” were “analyt*”, “barrier*”, and “educ*”, indicating an understanding that more development and user education are needed. This sentiment is exemplified in the response:

“*I find that the lack of accessible, effective visual analytics means many decision makers lack the ability to understand and interpret the data and make decisions informed by the data. I find that many policy and planning processes are based more on anecdotal data and subjective “understandings” of service systems rather than data, even where data exists (noting that data collection in mental health is often inconsistent in both quality and extent).*”

### Quantitative analysis

χ^2^ tests indicated that participants from different areas of work (e.g., policy, research or service provision) did not significantly differ in their ratings of the adequacy of currently available visual analytics for informing healthcare decisions, the importance of visual analytics for decision making, the need for new visual analytics tools for decision making, or their willingness to learn these new tools (Table 2). Similarly, perceived data complexity was not significantly associated with expectations of visual analytics as a tool for complex mental healthcare system planning (Table 2). This may suggest that there are no differences in expectations depending on what area the experts work or how complex they perceive their data to be. An important caveat is that the analysis is only powered to detect comparatively large differences between groups.

**Table 2:** Statistical associations of area of work and data complexity with expectations of visual analytics

	Area of work	Data complexity
Analysis	χ^2^	χ^2^
Dependent variable		
Most applied method	7.84 (0.90)	19.95 (0.65)
Adequacy to inform healthcare decisions	11.83 (0.50)	7.95 (0.81)
Importance of VA for decision making	12.03 (0.68)	10.71 (0.19)
New VA tools needed for analyses	12.02 (0.06)	14.62 (0.24)
Willing to learn new VA tools	3.11 (0.91)	15.12 (0.23)

Note. *VA* Visual Analytics. Values in brackets are significance. To account for sample size, the significance values are Monte Carlo simulated ‘*p*’ values at 10,000 replicates

## Discussion

This exploratory study has uncovered several promising avenues for further research and development of new and advanced visual analytics methods. The study results have demonstrated that experts in mental healthcare systems are aware of the importance of visual analytics. However, the health experts revealed that their approach to visual analytics was generally restricted to descriptive analysis with simple and familiar visualisations. Lack of understanding and low user-friendliness of advanced tools appeared to be major barriers to their application in the process of decision-making. Consequently, our results showed a demand for further co-development of effective and accessible visual analytics tools of practical benefit to mental healthcare policy planning. Further quantitative analysis revealed that these expectations do not largely differ on the basis of area of work (e.g., policy, research, or service provision) or data complexity. This suggests that the development of a general approach for engaging with health experts should be favoured in order to develop better visual analytics methods and increase their use. Thus, further work may benefit from focusing on expert understanding and needs, which can be achieved through co-development of both tools and skills.

User-friendliness was an underlying theme for health experts in both their current experience and requirements for the development of future visual analytics tools. This theme is vital to overcome the major barriers to investment in information parsing and decision-making processes, including time pressure [10], less developed expertise [25], poor visual literacy [26], and low trust in the data, analysis, or software developers underlying visualisation [27]. Thus, a co-development framework can help to achieve user-friendliness in the development of visual analytic tools. Fisher and Green [28] provided such a framework for the co-development of visual analytics in translational cognitive science and highlighted the applicability of the approach across projects and disciplines. There is some preliminary evidence of the benefits of this approach in practice, e.g., Freebairn and Atkinson [27] developed a dynamic simulation model for physical health planning and noted the co-development process led to greater expert uptake of the tool.

The mental health experts indicated working with complex data, yet predominantly reported using relatively simple descriptive statistics methods for their data analysis. They also rated these methods using basic graphs, charts, and geographical maps as highly applicable, acceptable, practical, and efficient. This may be symptomatic of the predominance of descriptive analysis applied by our sample driving reliance on simpler visualisation methods integrated into commonly used software such as Microsoft Excel, a software trend that has been observed in the context of disease epidemiology [29]. It is possible that while the data and its information processing are complex, the key information can be adequately visualised using familiar motifs (e.g., error bars are visually the same regardless of whether they are calculated via parametric or comparatively complex non-parametric iterative approaches). Most likely, the inherent reduction of complexity necessitated by simpler visualisations may meet the needs of speed of interpretability [10] and user-friendliness, at the cost of obscuring multidimensionality and hidden pattern information available in the underlying data [30]. Consistent, structured design and education to expand visual literacy can be effective in increasing expert engagement with more advanced visualisations and visual analytics tools [1]. However, such strategies have yet to achieve a widespread impact on expert utilisation of more complex approaches. For a shift to match complex data with more information-dense and non-standard visualisations, there is a need of further research on the degree of information loss from expert preference for visual simplicity and consequences for how decisions are made.

This study has a number of limitations. Healthcare systems expertise was a restrictive participation criterion. A more comprehensive scope not limited to mental healthcare would have provided a broader perspective on the experts’ perceptions on visual analytics and tools. However, it is important to consider that mental healthcare is at the core of integrated, multi-sectoral and complex approaches in healthcare systems research [31, 32], thus is a start point for this exploratory research. While representing a strong cross-section of senior mental healthcare systems perspectives (e.g., policy, provision and research) in Australia and in Europe, the response rate and small sample size limit generalisability. A reliance on self-report is vulnerable to recall bias and precludes more detailed investigation of participant engagement with visual analytics. However, the low response rate is a common problem to the majority of on-line surveys, and low response rates - generally above 20% - have demonstrated consistent and accurate results in previous studies [33, 34]. Although the sample size was sufficient for qualitative analyses (e.g., [35]), our quantitative comparison across area of work and degree of data complexity could only detect large effect sizes. This does suggest the absence of major differences between experts in policy, research, and service provision. Future research is required to establish whether there are subtle but meaningful differences in the experiences and expectations of experts depending on their area of work and complexity of data they work with.

## Conclusions

This study examined the perceptions and the real-world experience that experts in mental healthcare planning have with visual analytics tools. We investigated how well current visualisation techniques meet the expert needs and established suggestions for future development of visual and decision analytics, using a targeted online survey and combination of qualitative and quantitative analysis. Notably, this study was the first examination of self-report expert experience on the topic of using visual analytics in mental healthcare systems research and policy. Our preliminary findings indicated that, despite a clear need to extract information from highly complex data, experts tend to utilise visualisations that are most familiar to them, widely understood, and not necessarily the most appropriate. This undermined full utilisation of the depth of available evidences and risks leading to a shallow basis for mental healthcare decision-making. Positively, we found that mental health experts are aware of the value of visual analytics and open to further developments to enhance their applicability. We suggest that co-development may be the most fruitful approach in future research intended to develop effective and useful visual analytics tools for complex mental healthcare systems data.

## Supplementary information

**Additional file 1.** The online survey questionnaire used via Qualtrics platform for the data collection of this study.

## Data Availability

The datasets used and analysed during the current study are available from the corresponding author on reasonable request.

## Abbreviations

VIDEA: VIsual and DEcision Analytics
PECUNIA: ProgrammE in Costing, resource use measurement and outcome valuation for Use in multi-sectoral National and International health economic evaluAtions
VA: Visual Analytics
