# Supplementary material for: Experts’ perceptions on the use of visual analytics for complex mental healthcare planning: an exploratory study
Source: BMC Med Res Methodol. 2020 May 7;20:110. doi: 10.1186/s12874-020-00986-0 (PMC7206783; doi:10.1186/s12874-020-00986-0)
Supplement: Supplementary file 1 — Additional file 1. The online survey questionnaire used via Qualtrics platform for the data collection of this study. [file 12874_2020_986_MOESM1_ESM.pdf]

Supplementary materials

The Questionnaire in full (from Qualtrics)

Section 1. Participant Information

PLEASE NOTE: If you wish to be anonymous, do not complete this page and simply progress to the next page. The participant information if provided will be used for ACKNOWLEDGEMENT in our study outputs.

Full Name: [Given name Surname] (e.g. Florence Nightingale)

____

Position/Role: (e.g. CEO, researcher, planner, etc)

____

Area of work: (e.g. service provision, policy planning, etc)

____

Years of experience working in the healthcare domain:

____

Institution

____

Country

____

Section 2. Mental Healthcare Systems & Data

Mental healthcare systems are the structures that work to provide mental health care to citizens in defined areas, from policy to community clinics. They consist of all the organisations, people and actions involved in maintaining, restoring and enhancing human well-being.

Mental healthcare systems data is the information generated by these structures. It includes things such as service distribution and management, as well as service demand and individual outcomes. It can be used to monitor, evaluate, and plan for the future of mental healthcare systems.

What kind of mental healthcare systems data have you been involved with? (select all that apply)

- [ ] Types of services and availability (e.g. hospital, community centre, etc)
- [ ] Facilities and placement capacity (e.g. beds)
- [ ] Workforce capacity (e.g. psychiatrist, psychologist, nurse, etc)
- [ ] Societal information (e.g. social history, form of government, etc)
- [ ] Demography (e.g. age, sex etc)
- [ ] Cost (including insurance)
- [ ] Resource utilisation (e.g. admission rate, discharge status, length of stay, etc)
- [ ] Network and location
- [ ] All of the above
- [ ] Other (please specify) ____

What aspects of working with mental healthcare systems data have you been involved in? (select all that apply)

- ☐ Data collection
- ☐ Analysis
- ☐ Application to Policy or Planning
- ☐ All of the above
- ☐ Other (please specify)____

In the course of working with your mental healthcare systems data, what **analytical approaches or models** have you directly experienced? (select all that apply)

- ☐ Descriptive analysis (e.g. means, medians)
- ☐ Statistical analysis (e.g. linear models, ANOVA)
- ☐ Pattern analysis (e.g. machine learning)
- ☐ Geospatial analysis
- ☐ All of the above
- ☐ Other (please specify)____

What have you used analytical approaches in your mental healthcare systems data for? (e.g. the relationship/pattern between the number of beds and discharge/length of stay)

____

What **decisions or outcomes** have been informed by your work with mental healthcare systems data? (select all that apply)

- ☐ Admissions
- ☐ Discharges
- ☐ Professional provision
- ☐ Resource allocation (other than professionals)
- ☐ Future planning
- ☐ Accountability and reporting
- ☐ Policy guidance
- ☐ All of the above
- ☐ Other (please specify) ______________________________

How do you define **'complexity'** in the data you work with?
Note. The order of this rank list was randomised.

Click and drag these elements to rank them **from most to least** central to your own personal definition of data complexity.

______ Number of variables (more complex = more measures)
______ Structure (more complex = more nested elements e.g. patients within practises within catchments)
______ Variety (more complex = multiple data types e.g. self-report survey, admissions, prescriptions)
______ Size (more complex = larger number of individuals, cases, or data points available)
______ Contributors (more complex = more sources of data, e.g. doctors from multiple sites, policymakers, and patients)
______ Relationships (more complex = more interactions between elements, e.g. seasonality, world events, clinic capacity)
______ Abstraction (more complex = further away from raw measures (such as MRI scans and cognitive tests) and closer to interpretation (e.g. policy decisions based on Dementia diagnosis from that data))
______ Uncertainty and ambiguity (more complex = more uncertain and/or ambiguous)
______ Difficulty of prediction or forecasting (more complex = more difficult)

Based on this ranking, how would you rate the complexity of mental healthcare systems data you have worked with (in general)?

- ○ 1 (Very simple)
- ○ 2
- ○ 3 (Neutral)
- ○ 4
- ○ 5 (Very complex)

Section 3. Visual Analytics

Visual analytics is the suite of tools that use visualizations to combine automated analysis techniques (e.g. machine learning algorithms) with human expertise. This is done to capitalize on complex data, and drive effective understanding and decision-making.

Which of the following **visualisations** have you applied to **mental healthcare systems data**? (select all that apply)

- ☐ I have not used any visualisation tools for my mental healthcare systems
- ☐ Basic graphs or charts (e.g. bar, line, pie, spider, scatter, etc)
- ☐ Geographical maps
- ☐ Network graphs
- ☐ Wheel graphs
- ☐ Rose diagrams
- ☐ Tree diagrams
- ☐ Icon plots
- ☐ All of the examples
- ☐ Other (please specify) ____

Which of the following visualisation tools have you had the **most preferred** working with?

- ◯ I have not used any visualisation tools
- ◯ basic graphs or charts
- ◯ geographical maps
- ◯ network graphs
- ◯ wheel graphs
- ◯ rose diagrams
- ◯ tree diagrams
- ◯ icon plots
- ◯ other ____

What did you use this visualisation tool for? (one or two sentences)
(e.g. line graphs may be used for analysing a time trend)

____

Thinking about your experience using visualisation tools, how would you rate:

	1 (Very poor)	2	3 (Neutral)	4	5 (Excellent)
**Applicability** (the degree to which the visualisation is meaningful for analysis and subsequent decisions)	○	○	○	○	○
**Acceptability** (user-friendliness and likelihood of uptake)	○	○	○	○	○
**Practicability** (ability to effectively implement and interpret)	○	○	○	○	○
**Efficiency** (capacity to clearly summarise large and complex data or model results)	○	○	○	○	○

In your opinion, are the **currently available or experienced** analytical approaches or models adequate to inform the decisions being made, given available mental healthcare systems data?

- ◯ 1 (Extremely inadequate)
- ◯ 2
- ◯ 3 (Neutral)
- ◯ 4
- ◯ 5 (Extremely adequate)

How **important** are visual analytics tools in your field?

- ◯ Unimportant or not used at all
- ◯ Neutral (Neither unimportant nor important)
- ◯ Important

Do you think that more of new and advanced visual analytics tools are needed for your analysis?

- ◯ Yes
- ◯ Maybe
- ◯ No

Are you willing to learn **new and advanced** visual analytics tools for understanding more complex data?

- ◯ Yes
- ◯ Maybe
- ◯ No

This is the **final question**. Before you submit, do you have any further **comments on the topic of visual analytics for mental healthcare data** that you would like to add?

____

____

____

____

____
